# Experience‐based co‐design—Adapting the method for a researcher‐initiated study in a multi‐site setting

**DOI:** 10.1111/hex.13028

**Published:** 2020-02-11

**Authors:** David K. Raynor, Hanif Ismail, Alison Blenkinsopp, Beth Fylan, Gerry Armitage, Jonathan Silcock

**Affiliations:** ^1^ University of Leeds Leeds UK; ^2^ University of Bradford Bradford UK

**Keywords:** adapted method, codesign, complex intervention, experience‐based co‐design

## Abstract

**Background:**

Experience‐based co‐design (EBCD) brings patients and staff together to co‐design services. It is normally conducted in one organization which initiates and implements the process. We used the traditional EBCD method with a number of adaptations as part of a larger research study in the British National Health Service.

**Methods:**

The primary aim was to assess the feasibility and acceptability of conducting research‐initiated EBCD, to enhance intervention development prior to testing. As well as embedding the method in a research study, there were 3 further key adaptations: (a) working across primary and secondary care sectors, (b) working on multiple sites and (c) incorporating theory‐informed analysis.

**Results:**

We recruited four sites (covering both primary and secondary care) and, on each site, conducted the initial traditional EBCD meetings, with separate staff and patient groups—followed by a single joint patient‐staff event, where four priority areas for co‐design were agreed. This event was driven by theory‐informed analysis, as well as the traditional trigger film of patient experiences. Each site worked on one priority area, and the four co‐design groups met over 2‐3 months to design prototype tools. A second joint event was held (not usually undertaken in single‐site EBCD) where they shared and compared outputs. The research team combined elements of these outputs to create an intervention, now being tested in a cluster randomized controlled trial.

**Conclusions:**

EBCD can be successfully adapted for use across an entire patient pathway with multiple organizations and as part of a research process to identify an intervention for subsequent testing in a randomized trial. Our pragmatic approach used the patient experience to identify areas for improvement and co‐designed an intervention which directly reflected patient priorities.

## 1 BACKGROUND

### 1.1 Experience‐based co‐design in health service improvement and research

Experience‐based co‐design (ECBD) has been shown to be a powerful tool for developing health service improvements—and acceptable to both service users and staff 1, 2, 3 ^.^ Based on a narrative‐based participatory approach, the method is designed to lead to service improvements by bringing together staff and service users to collaboratively co‐design services.^4^ Since the first reported use of EBCD in 2007,^5^ a 2014 review identified over 59 projects in six countries—usually within a single health‐care organization.^6^ At the centre of the method is a ‘trigger film’ of patients talking about their experiences to stimulate joint discussions between patients and staff around service delivery issues. These discussions focus on identifying priorities for improvement and are followed by co‐design groups to develop prototype solutions. Such videos can be highly effective, facilitating collaboration of participants with different perspectives.^7^ EBCD was first described in a ‘tool kit’ from the Kings Fund and is now housed at the Point of Care Foundation.^8^ Health service researchers have recognized the importance of co‐designed interventions; however, at the time of our study (2016), there were no published studies that reported using EBCD in intervention development for evaluation in a randomized control trial (RCT). Therefore, our paper makes a novel and timely contribution to the further development of participatory methods.

### 1.2 Adapting the EBCD method

A particular appeal of EBCD is the ability to adapt it to particular circumstances. With any such adaptations, the importance of retaining the patient interviews and interaction between patients and staff has been emphasized.^6^ Whilst designing a study to develop an intervention to be tested in a randomized controlled trial, we identified that as well as being a service improvement tool, EBCD could also be powerful as an integral part of the research process to help identify a patient‐centred intervention. Hence, we designed and operationalized an adapted EBCD as a key part of intervention development. This paper, therefore, describes a study within a larger study.

Our novel approach also included:

- working with a group of 9 British National Health Service (NHS) health‐care providers (including both primary and secondary care sectors) covering four distinct geographical areas (each with a population of between ~450 000 and 750 000). In this paper, we will describe each of these four sites as ‘health‐care economies’.
- undertaking the process on four sites—rather than the usual single site
- augmenting the trigger film through systematic analysis using resilience and systems theories (drawing on data collected in a preceding mixed‐methods study—observations, document analysis and interviews together with a systematic review^9^).

### 1.3 Developing an intervention for managing medicines at transitions of care

The focus of our study was addressing the problems associated with how medicines are managed at transitions of care—specifically when cardiology patients are discharged from hospital.^9^ Despite very strong evidence of effectiveness of medicines in heart failure, outcomes for heart failure continue to be sub‐optimal and survival rates have not improved in line with other health conditions.^10^ We found no previous studies that had used EBCD to explore medicines management for heart failure patients*.* Our planned RCT was to be conducted in 42 areas of England, and the engagement of multiple sites was critical to intervention development. After considering a range of co‐design methods, we concluded that an EBCD process adapted for multiple sites would best meet the needs of the larger study.

### 1.4 Aims and objectives

This paper aims to assess the feasibility and acceptability of health service researchers co‐leading EBCD in multiple health‐care settings as part of intervention development.

The objectives are to:

1. Examine the feasibility of implementing the EBCD process in a health service across health‐care organizations on different sites and in different settings (both primary and secondary care) and integrating existing theory
2. Determine the acceptability of patients, researchers, quality improvement (QI) leads and clinicians working together to develop an intervention.
3. Identify the challenges for different participants specific to their ambitions, organizational processes or outcomes.

## 2 METHODS

### 2.1 EBCD in the ISCOMAT study

The EBCD was embedded within the design of a wider UK programme of research:

- This research was leading to a large national randomized controlled trial of an intervention.
- ‘Improving the Safety and Continuity of Medicines management at Transitions of care’ (ISCOMAT) was designed to study medicines management for patients with heart failure across a range of health organizations involved in their care in the National Health Service (NHS).^11^
- The focus is on working with patients and health‐care staff to develop and test a toolkit to improve the management of medicines when patients move between hospital and home.

**Figure 1:**
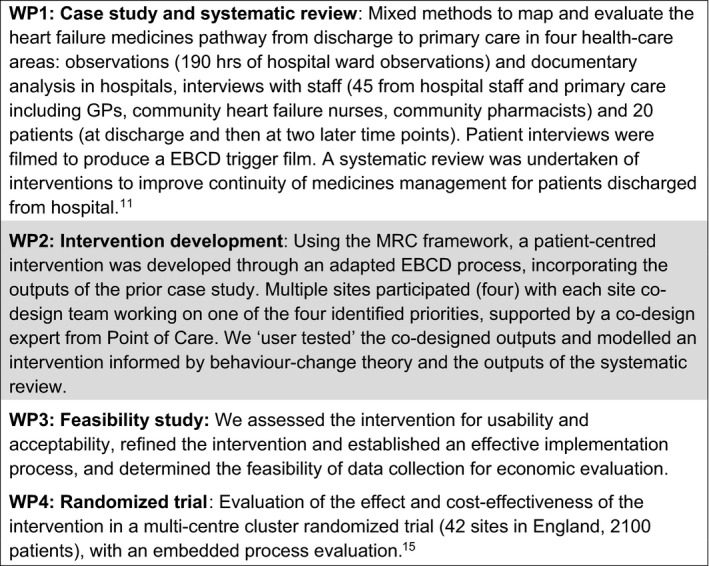
ISCOMAT study summary showing the context of the EBCD

Figure 1 shows the ISCOMAT study summary showing the context of the EBCD.

### 2.2 The EBCD Procedure

**Figure 2:**
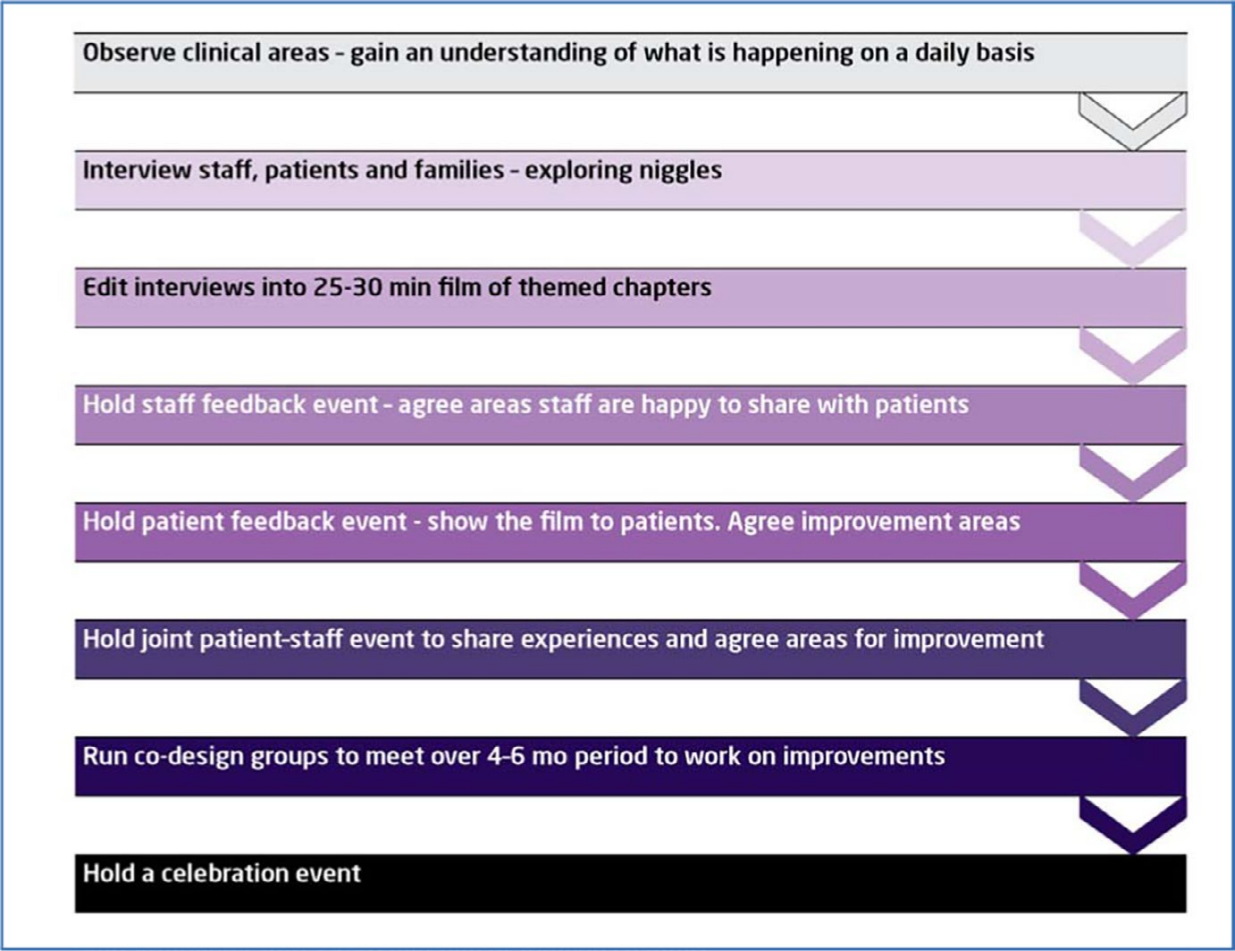
Steps in the traditional EBCD process

We drew on previous work undertaken by the King's Fund and others to adapt the EBCD process.^12^ The steps in the traditional EBCD process are listed in Figure 2:

In order to reflect diversity, we ensured that the 4 sites chosen represented a mix of urban, rural and coastal locations and consisted of both large teaching hospitals and smaller district general hospitals. All sites contained both deprived and affluent areas, and three were in areas that contained ethnically diverse populations. The sites were located within a 60 mile geographical radius from the research team's base to facilitate data collection and minimize the travel burden for both health professionals and patients.

For each of the four sites invited to take part in the EBCD, the research team established the internal organizational process for quality improvement (QI) to enhance organizational benefit through the potential for collaborative working. The team then approached the lead for that process to secure engagement. Training on conducting EBCD was delivered by the Point of Care Foundation for the:

- research team
- ISCOMAT patient‐led steering group members and
- sites’ quality improvement leads

Following this, a local champion was identified for each of the four sites. These comprised: site 1: quality improvement manager; 2: hospital cardiology pharmacist; 3: research nurse; and 4: cardiologist.

An earlier work package in the larger study had recruited the four sites as research settings, and we invited all patients who had been interviewed about their experiences of receiving heart failure medicines to take part in the EBCD process (see ‘theory‐informed analysis’ below). Sixteen patients and family members, and 24 staff members participated in the patient and staff meetings at the four sites. Table 1 lists the patient event participants and Table 2 the staff event participants. The joint event was co‐facilitated by a patient representative and a professor of cardiovascular health, with support from an expert in EBCD and a researcher working with the ISCOMAT patient‐led steering group. It was attended by 34 participants (17 patients and carers, 13 health professionals and four others). Table 3 lists the participants of the first joint event. It was co‐chaired by a patient and a consultant cardiologist. In addition, 11 research team members were in attendance.

**Table 1:** Patient event participants

	Patients	Family members/carers	ISCOMAT PLSG members
Site 1	2	1	
Site 2	3	2	
Site 3	2	2	1
Site 4	3	1	1

**Table 2:** Staff event participants

	Specialist HF nurses	GPs	HF consultant	Pharmacists	Other
Site 1	3	1	1		1 (Palliative care consultant)
Site 2	1	2	1	1	2 (Cardiology Business Manager; Research nurse)
Site 3	2	1		1	4 (NHS Trust Quality Improvement Manager; CCG: Senior Commissioning Manager; Quality Manager; Senior Quality Officer)
Site 4	1			2	

**Table 3:** First joint event

	Patients	Family members/carers	Health professionals	Others
Site 1	2	1	3	‐
Site 2	3	2	4	‐
Site 3	‐	‐	3	1 (Commissioning Pharmacist)
Site 4	2	2	2	2 (Quality Manager & Project Officer)
Patient Led Steering Gp	5			
CCG			1	1 (Head of Research)

Note

The first joint event was attended by 11 research team members, 2 invited academics, a guest co‐presenter (Professor of Cardiovascular Health) plus the following 34 participants.

The 20 patients and 45 staff who had participated in the four case study areas were invited to the separate EBCD events for patients and for staff—at each of the four sites. Events were facilitated by two members of the research team with previous experience of working with patients and health‐care professionals. An external facilitator (HB) from the Point of Care Foundation who advised on event design was present at the events and provided design support to the co‐design groups. In producing the trigger video, the research team worked with the patient‐led steering group, notably its chairperson.

Our EBCD included three key adaptations:

1. Working across health‐care organizations and between four health‐care economies—EBCD generally takes place in one organization, whereas here both hospital teams and primary care (including general practitioners, pharmacists and heart failure nurses) are critical to successful medicines management in heart failure and were included in the co‐design process.
2. Working with multiple sites—Traditionally, EBCD takes place within the confines of one site. However, we were aware that different models for managing heart failure and associated medicines existed across NHS local systems and set out to capture this within intervention development. In the previous case study, we purposively selected four health economies and within a 60‐mile catchment area of the most centrally located site. Patient and staff EBCD meetings were held at each of the four sites. A feedback summary was produced from each of these eight meetings, and we then held a multi‐site joint patient/staff meeting that considered the insights and areas for opportunity that had emerged.
  Participants then generated a long list of priorities relating to areas that could be improved and then agreed key priorities to take forward in the co‐design process. This was an important adaptation to the traditional EBCD approach, where traditionally one design solution would apply to a single site. After the joint meeting, the co‐design work was undertaken by four local groups (each led by the local site champion) who led the development and prototyping of tools to form part of the intervention, supported by the experienced Point of Care Foundation facilitator. Finally, we reconvened the multi‐site meeting, bringing co‐design group members together to present their results to each other.
3. Incorporating theory‐informed data analysis**—**Traditionally, EBCD involves some observations of the delivery of care and interviews with patients and staff. The quantity of data collected is variable and often subject to local resource constraints. Our research study allowed for a larger data collection and analysis exercise which included ward observations and staff and patient qualitative interviews. We adopted a ‘Safety II’ approach which acknowledges that despite a considerable focus on learning from adverse events in health care, achieving extensive improvements in the safety of care has been slow.^13^ Safety II accepts the complexity of health care and prioritizes learning from the safe delivery of care to patients and how they can play a key role in their own safety.^14^ We were able to present our analysis to patients and staff at our separate and joint events to inform their view of the system and the positive roles they played within it, whilst highlighting the potential for safer care through system modification.^9^ Staff, in particular, were receptive to the positive perspective of Safety II, which demonstrated how they and their colleagues were flexible and adaptable in the face of pressure to promote safer outcomes for patients, even where their actions were not necessarily mandated or recognized by their organizations.

**Figure 3:**
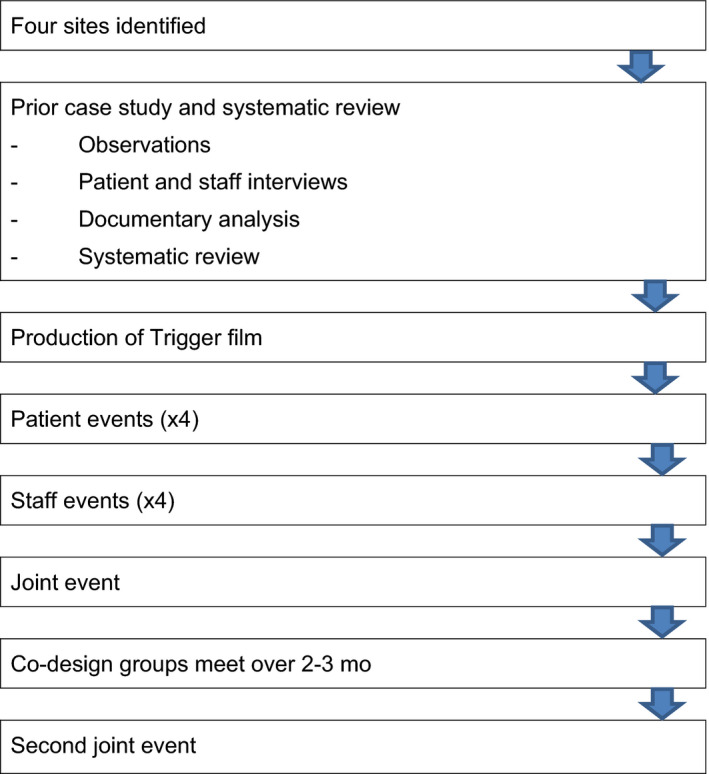
Flow chart for the adapted EBCD method

Figure 3 shows the adapted EBCD process used in this study in flow chart format.

## 3 RESULTS

### 3.1 Feasibility and acceptability of process to participants across health‐care organizations and in multiple sites

**Figure 4:**
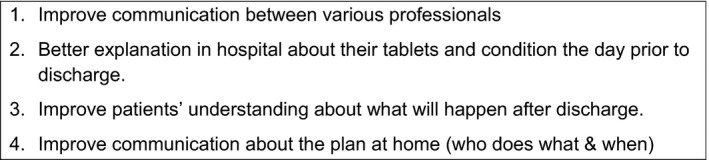
Priorities agreed at the first joint patient/staff meeting

Having successfully negotiated with the four sites, all of which agreed to participate in the EBCD project, the patients and staff recruited took part in patient and staff meetings at each site. At the joint meeting, feedback presentations were used to explore insights and areas for opportunity for improvement. The participants generated a long list of 15 priority areas which they then grouped and narrowed down to their top four. Figure 4 lists the priorities jointly agreed at the first joint patient/staff meeting. This showed that having patients and staff working together on multiple sites—and then coming together at a joint meeting—could successfully generate priorities for the subsequent co‐design work.

As part of the joint meeting, it was agreed which site would work on which priority and the members of the respective co‐design teams for each site were identified. In total, 12 patients and 16 staff members were part of the local co‐design teams. Each team consisted of four to seven individuals, who were asked to develop a tool that would address their team's priority and they met at their individual sites over a 2‐ to 3‐month period. The teams identified opportunities to improve the service or the care pathway related to their priority area and designed prototype ‘tools’.

Finally, the tools designed at each site were shared in a second central joint meeting of staff and patients from the four areas together with members of the research team. The second joint event was attended by 15 participants (10 patients and carers, four health professionals and a quality manager), along with 6 members of the research team. Table 4 lists the participants in the second joint event.

**Table 4:** The second joint event was attended by 6 research team members plus the following 15 participants (10 patients and carers and 5 staff members)

	Patients	Family members/carers	Health Professionals	Others
Site 1	1	1	1	‐
Site 2	1	‐	1	‐
Site 3	‐	‐	1	‐
Site 4	2	2		1 (Quality manager)
CCG			1	‐
Patient Led Steering Gp	3			

Such a second joint meeting does not normally take place in single‐site EBCD. However, as our co‐design teams were working separately on tools to address different priorities within the care pathway, the multi‐site EBCD processes required an additional event to enable the teams to share and compare their outputs, along with any plans they had to implement their tools locally. We also observed during this second event how the separate teams took pride in presenting their tools to the larger group and how they enjoyed further contact with those they had shared their ideas and experiences with at the earlier joint event. Our informal observations at each event enhanced our view that invited participants were fully engaged in the process. The joint conversations suggested that the various team members—representing disciplines and roles that do not routinely have the opportunity to dissect the medicines management process—valued the protected time and space to work on practical and pressing challenges, but significantly, *with* patients.

Our records of attendance served as a proxy measure of acceptability concerning the EBCD process.

#### 3.1.1 Use of theory‐informed data analysis to support the EBCD process

The trigger film drew upon 55 interviews with 20 patients (at discharge, then 2 and 6 weeks later). In addition to the trigger film, a presentation which summarized key findings from the prior case study was included at the first joint event, based on a resilience‐informed analysis and including the findings from the systematic review. The presentation followed the patient journey and detailed what makes the system more and less resilient. The case study identified variations in the way health care was structured and delivered in the four areas. For example, specialist heart failure nurses performed domiciliary visits in some areas but not in others, and due to staff availability, the typical waiting period for the first visit post‐hospital discharge ranged from 21 days to 12 weeks. The data in this presentation were then successfully incorporated into the discussions which followed.

After the second joint event, the research team were able to combine elements from each of the outputs to create an intervention for heart failure patients at discharge from hospital. The creation of the intervention and its use within our RCT are described in detail elsewhere,^11^ but briefly the ‘Medicines At Transitions Intervention’ consists of:

1. Online training to secondary care cardiology, community pharmacy and primary care staff about discharge management (including medicines).
2. Patient‐held information.
3. Delivery of education to patients about their medicines.
4. Enhanced communication between hospital and the patients’ community pharmacists
5. Increased engagement of community pharmacists with patient care after discharge

#### 3.1.2 Integrating with local quality improvement processes

Processes for approving and leading quality improvement varied across the four sites. For example, in a large teaching hospital, responsibility for innovation was devolved to clinical directorates, but in a district general hospital, a central panel approved changes and had a fixed, multi‐step process. Patient engagement and structured facilitation were key to ensuring the co‐design process was effective when working with service improvement leads and alongside existing processes or initiatives designed to deliver quality improvements and drive transformational change. The EBCD process was particularly well received in one site, as it enabled them to achieve a national target of increasing patient engagement.

After identifying and engaging a key local decision‐maker and agreeing on the EBCD process, sites took on board the relevance and potential value of the project. The local champions’ participation in joint EBCD training with the research team helped to build rapport and working relationships between NHS organizations and the research team and contributed to organizational development for service improvement teams.

### 3.2 Challenges

We faced a number of challenges in planning and implementing the EBCD process in the partnering sites. These ranged from the purely logistical, for example finding suitable locations to hold events and developing focussed activities to engage both patients and health professionals in purposeful conversations.

Other challenges involved retaining the interest of both parties (service users and health professionals) over a sustained period and managing expectations and outcomes; EBCD projects are often expected to deliver tangible quality improvement outcomes quickly and cost‐effectively.^15^ Aligning patients’ expectations and motivation to improve their lived experience of the service with health professionals’ work routines and their need to implement measurable quality improvements also proved challenging. We addressed this by recognizing the inevitable ‘power imbalance’ that exists within EBCD and by championing the voice of the service users by ensuring that our whole approach hinged on a patient‐centred redesign process.

## 4 DISCUSSION

By using EBCD methods in multiple locations, the input of patients and providers was able to support patients and staff to agree to a common set of priorities with respect to medicines management in heart failure patients. Despite differences in the local care pathways for patients discharged from hospital with heart failure, patients and staff were able to identify key priorities that applied across all four of the NHS patient pathways. Furthermore, although sites were allocated different priorities to work on, they valued the opportunity to come together and learn from the experiences of other sites.

It has been noted that practical advice on how and when to use EBCD is limited^16^ and our findings fill some of these gaps, notably that it is feasible and acceptable to conduct research‐initiated multi‐site EBCD to enhance intervention development in a study of heart failure medicines management. This reframing of the process resulted in the creation of an intervention which is currently being tested in a randomized trial. We used EBCD to help us understand what needed to change and to develop some co‐designed prototypes for how these changes could be brought about. We argue that multi‐site co‐production methodologies can enhance intervention development and provide a mechanism to translate available evidence into patient‐centred, intervention proposals.

Our adaptations to the traditional EBCD approach yielded unexpected benefits and created a clear process for co‐learning. Our inclusion of a second joint meeting to share and discuss the prototype tools developed by the co‐design groups enabled them to share their findings, and we suggest this additional step is essential in multi‐site EBCD. Richard et al noted that engagement is essential in trials’ research but rarely embedded across all stages of the research continuum.^17^

Our work has identified issues related to researchers engaging with participating organizations’ processes for approving and leading innovation, including NHS quality improvement leads. We encountered several challenges in organizing and conducting the EBCD process which have been noted by other researchers: power to authorize change, commitment to the process, methods for gathering experiences, designing improvements, implementation and subsequent impact.^7^, ^15^ At each site, we explored the hospital's own process for service improvement and the individual(s) that made decisions. We found that quality improvement structures, processes and responsibilities were much clearer in NHS hospitals than in their adjoining primary care settings. As our intervention was initiated in the hospital setting, this meant we were able to work within the existing systems. Organizational commitment and internal power were considered through our initial identification of an individual with the power to grant authorization for the EBCD process. The local champions provided organizational commitment, and their enthusiasm and drive were critical in the co‐design groups’ completion of their tasks. For any EBCD project to be successful, it should be championed by individuals who are strong leaders and have the authority to drive change in their organizations.

The collection and analysis of local data by the research team removed a key barrier in EBCD by providing resources for robust and systematic data. The collection and analysis of local data by the research team removed a key barrier in EBCD by providing resources for robust and systematic data. However, although sharing these data with local teams was undoubtedly powerful, some patients and staff may have felt that their original thinking was somewhat primed. The contribution of the research team in facilitating EBCD cannot be underestimated, as the sites we worked with had no previous experience of using EBCD to engage with patients. We not only introduced organizations to EBCD, but we also guided them through each stage and undertook the most challenging part of the process—the recruitment of patients and the creation of the trigger film. We found that local quality improvement teams did become engaged in the process, and the research team coordinated their involvement across the four areas.

In the future, local teams might themselves collaborate across sites in EBCD. Oversight and coordination might work through existing networks—for example in the NHS via Academic Health Science Networks^18^ which bring together British universities and NHS health‐care providers to innovate at pace and scale at regional level. Our trigger film is now in use nationally in a patient safety programme across networks. Such systematic collaboration might also facilitate shared QI initiatives at a system level in Integrated Care Partnerships,^19^ a current policy priority across the NHS in England. Perhaps the key message is that researchers and QI teams could collaborate and build this in from the research planning stage. This might be particularly helpful in patient safety studies, a topic that has a well‐established relationship with QI.

Unusually for a study using co‐design in intervention development, our project included all five key EBCD elements:

1. inclusion of the full range of stakeholders,
2. involvement of users throughout the study,
3. inclusion of the whole service,
4. focus on user experience and
5. consideration of the implications for the interface between users and service providers.

We found only one published study of the use of co‐design in intervention development for heart failure management and that utilized ‘expert’ health‐care professionals known to the researchers rather than those involved in delivering care in the organization from which patient participants were recruited.^20^ Richard et al reported that relatively few studies report on the engagement of public, patient and/or service users across the research continuum.^17^ Our paper makes a valuable contribution to sharing methods of active joint working between researchers, NHS patients and staff, beyond simply collecting data to co‐producing a new intervention.

Clarke et al have noted the lack of rigorous evaluation of the impact of EBCD in acute health‐care settings and recommended that future studies should evaluate clinical and service outcomes.^21^ Only one feasibility RCT evaluating the impact of a co‐designed intervention to improve the knowledge, experience and emotional well‐being of carers of people with breast, lung or colorectal cancer reported the use of a validated outcome measure, the General Health Questionnaire.^21^

### 4.1 Limitations

Informal discussions with the two sites that continued, after the EBCD, to offer participation in the RCT feasibility study have since indicated that neither had adopted the intervention beyond the study period itself. In this respect, although the EBCD was successful as a method of intervention development it seems to have had little impact on practice. Dimopoulos‐Bick et al in their review note that there is little evidence about the wider impact of experience systems.^15^ We had hoped that the process would have led to lasting changes at each of the sites—and although this did not occur, our experience suggests it is not indicative of any weakness in commitment during the EBCD process itself. Others who may wish to adopt this approach might spend more time securing and ratifying senior management support as part of the planning process. Additionally, we are uncertain about the feasibility of implementing this process in countries where local health economies lack a history of collaborative working.

## 5 CONCLUSION

EBCD has previously been shown to work in a single site and usually with a single organization. We have shown that EBCD can be successfully adapted for use across an entire patient pathway with multiple organizations and between health economies. Each team across the four sites delivered a new tool that addressed the concerns identified in priority areas for patients and staff.

Multi‐site EBCD is a novel and valuable collaborative approach to service improvement and has the potential to enhance the effectiveness of service development across organizational and physical boundaries. Our pragmatic approach in using EBCD is that we have not only used patient experience to identify areas for improvement but have co‐designed an intervention directly reflecting patients’ priorities to be tested in a cluster randomized control trial that seeks to provide evidence for or against adoption.

As in traditional EBCD, we showed that patients and health‐care professionals can work together to identify and implement changes that benefit both service providers and end users. Furthermore, enhancing communication between patients and health‐care professionals resulted in mutual learning and respect for each other's viewpoints. Moreover, it may help to erode the traditional power imbalance between the two parties.

## CONFLICT OF INTERESTS

DKTR is co‐founder and academic advisor to Luto Research which develops, refines and tests health information materials. The remaining authors declare that they have no competing interests.

## ETHICS APPROVAL AND CONSENT TO PARTICIPATE

The EBCD project did not require formal ethical approval as it was defined as a service improvement/PPI initiative—and it was agreed as such with the sponsor.

## Data Availability

The data that support the findings of this study are available on request from the corresponding author. The data are not publicly available due to privacy or ethical restrictions.

## References

[1] Bevan H , Robert G , Bate P , Maher L , Wells J . Using a design approach to assist large‐scale organizational change “10 High Impact Changes” to improve the National Health Service in England. Journal of Appl Behav Sci. 2007;43:135‐152.

[2] Bowen S , McSeveny K , Lockley E , Wolstenholme D , Cobb M , Dearden A . How was it for you? Experiences of participatory design in the UK health service. CoDesign. 2013;9:23046.

[3] Palmer V , Chondros P , Piper D , et al. The CORE study protocol: a stepped wedge cluster randomised controlled trial to test a co‐design technique to optimise psychosocial recovery outcomes for people affected by mental illness in the community mental health setting. BMJ Open. 2015;5(3):e006688. 10.1136/bmjopen-2014-006688 PMC4386225 25805530

[4] Bowen S , McSeveny K , Lockley E , Wolstenholme D , Cobb M , Dearden A . (). How was it for you? Experiences of participatory design in the UK health service. CoDesign. 2013;9:230‐246.

[5] Bate SP , Robert G . Towards more user‐centric organisational development: lessons from a case study of experience‐based design. J Appl Behav Sci. 2007;43:41‐66.

[6] Donetto S , Tsianakas V , Robert G . Using Experience‐based Co‐design (EBCD) to improve the quality of healthcare: mapping where we are now and establishing future directions. London, UK: National Nursing Research Unit 2014; https://www.kcl.ac.uk/nmpc/research/nnru/publications/reports/ebcd-where-are-we-now-report.pdf. Accessed January 13, 2020.

[7] Mulvale A , Miatello A , Hackett C , Mulvale G . Applying experience‐based co‐design with vulnerable populations: Lessons from a systematic review of methods to involve patients, families and service providers in child and youth mental health service improvement. Patient Exp J. 2016;3:117‐129.

[8] The King’s Fund . Experience‐based co‐design toolkit; 2012 http://www.kingsfund.org.uk/projects/ebcd. Accessed January 13, 2020.

[9] Fylan B , Marques I , Ismail H , Breen L , Gardner P , Armitage G . Blenkinsopp A on behalf of the ISCOMAT Programme Team. Gaps, traps, bridges, and props: a mixed‐methods study of resilience in the medicines management system for patients with heart failure at hospital discharge. BMJ Open. 2019;9(2):e023440. 10.1136/bmjopen-2018-023440 PMC6377507 30782879

[10] Taylor CJ , Ordóñez‐Mena JM , Roalfe AK , et al. Trends in survival after a diagnosis of heart failure in the United Kingdom 2000–2017: population‐based cohort study. BMJ. 2019;364:1223. 10.1136/bmj.l223 PMC6372921 30760447

[11] ISCOMAT trial summary. https://www.isrctn.com/ISRCTN66212970. Accessed January 13, 2020.

[12] Tsianakas V , Robert G , Maben J , Richardson A , Dale C , Wiseman T . Implementing patient‐centered cancer care: using experience‐based co‐design to improve patient experience in breast and lung cancer services. J Support Care Cancer. 2012;11:2639‐2647. 10.1007/s00520-012-1470-3 PMC3461206 22544223

[13] Braithwaite J , Wears RL , Hollnagel E . Resilient health care: turning patient safety on its head. Int J Qual Health Care. 2015;27:418‐420. 26294709 10.1093/intqhc/mzv063

[14] Mannion R , Braithwaite J . False dawns and new horizons in patient safety research and practice. Int J Health Policy Manag. 2017;6:685‐689. 29172374 10.15171/ijhpm.2017.115 PMC5726317

[15] Dimopoulos‐Bick T , Dawda P , Maher L , Verma R , Palmer V . Experience‐based co‐design: tackling common challenges. J Health Des. 2018;3:86‐93.

[16] Castro EM , Malfait S , Van Regenmortel T , Van Hecke A , Sermeus W , Vanhaecht K . Co‐design for implementing patient participation in hospital services. Patient Educ Couns. 2018;101:1302‐1305. 29602511 10.1016/j.pec.2018.03.019

[17] Richard L , Piper D , Weavell W , et al. Advancing engagement methods for trials: the CORE study relational model of engagement for a stepped wedge cluster randomised controlled trial of experience‐based co‐design for people living with severe mental illnesses. Trials. 2017;18:169 10.1186/s13063-017-1878-7. 28388937 PMC5385022

[18] https://www.ahsnnetwork.com. Accessed November 9, 2019.

[19] https://www.england.nhs.uk/integratedcare/integrated-care-systems/. Accessed November 8, 2019.

[20] Hjelmfors L , Strömberg A , Friedrichsen M , Sandgren A , Mårtensson J , Jaarsma T . Using co‐design to develop an intervention to improve communication about the heart failure trajectory and end‐of‐life care. BMC Palliat Care. 2018;17:85. 29890974 10.1186/s12904-018-0340-2 PMC5996457

[21] Clark D , Jones F , Harris R , Robert G . What outcomes are associated with developing and implementing co‐produced interventions in acute healthcare settings? A rapid evidence synthesis. BMJ Open. 2017;7:e014650. 10.1136/bmjopen-2016-014650 PMC5734495 28701409

